# Agronomic evaluation of microalgal extracts as seed bio stimulants for improving germination and early growth of common bean

**DOI:** 10.1038/s41598-026-58497-9

**Published:** 2026-06-25

**Authors:** Kholoud Osama Elhakim, Fatma Abd El Lateef Gharib, Eman Zakaria Ahmed

**Affiliations:** Botany and Microbiology Department, Faculty of Science, Helwan (Capital) University, Cairo, Egypt

**Keywords:** Common bean, *Phaseolus vulgaris*, Microalgal extracts, Germination, Seedling growth, Enzymes activities, Biotechnology, Physiology, Plant sciences

## Abstract

Microalgal extracts are rich sources of diverse bioactive primary and secondary metabolites with potential bio stimulatory effects. This study comparatively evaluated extracts from *Chlorella vulgaris*, *Nannochloropsis salina*, and *Arthrospira platensis* as natural seed-priming agents, aiming to identify the most effective species and optimal concentration for enhancing seed germination and early seedling development of common bean (*Phaseolus vulgaris* L.). Seeds were primed for 4 h in aerated algal extract solutions at concentrations of 0.0, 0.13, 0.25, 0.5, 1.0, 2.0, and 5.0%, followed by germination under controlled conditions (25–30 °C) for six days. Seed priming with algal extracts at concentrations up to 1.0% significantly improved germination percentage (GP), seedling vigor index (SVL), seedling length and biomass, and hydrolytic enzyme activities compared with untreated controls. The most pronounced stimulatory effects were observed at 0.5%, particularly with N. salina, which increased germination to 96% compared with 84% in the control, along with a ~ 130% increase in seedling vigor index. These enhancements were accompanied by elevated activities of hydrolytic enzymes and increased levels of total soluble sugars and proteins. Conversely, higher extract concentrations (2.0 and 5.0%) exerted inhibitory effects on germination and seedling growth, indicating a clear concentration-dependent response. Notably, S. platensis at 5.0% induced the largest decrease in GP (61%), along with a ~ 62.65% decrease in SVI.

## Introduction

Common bean (*Phaseolus vulgaris* L.) (Fabaceae Family) is one of the most important leguminous crops used for human nutrition, with a commercial value exceeding that of all other bean crops^1^. Dry beans are rich in dietary fiber, essential minerals, and resistant and slowly digestible starches, which together enhance their nutritional and health-promoting value^2^. Common bean germination is frequently hampered by various factors such as soil conditions, temperature, watering practices, seed health, and a variety of diseases. This problem can lead to reduced crop yields and poor seed quality. The use of chemical agents or bio stimulants can help mitigate these challenges and promote successful growth.

Bio fertilizers and bio stimulants represent sustainable agricultural inputs that enhance plant growth, nutrient use efficiency, stress tolerance, and disease resistance while reducing reliance on synthetic agrochemicals. Driven by regulatory restrictions and growing demand for organic crops, microalgae and cyanobacteria have emerged as effective bio-based growth promoters. Their beneficial effects are attributed to diverse bioactive compounds, including amino acids, micronutrients, polysaccharides, phytohormones, and other signaling molecules, which act synergistically to enhance crop productivity, yield, and quality in an environmentally sustainable manner^3–5^. This is done through various application strategies such as adding alive or dried algae biomass to the soil, treating seeds with microalgae cell extract, and applying the extracts directly to the roots^6^. Seed treatment with bio stimulants involves three different processes, namely seed priming, seed coating, and seed dipping^7^. The major benefit of utilizing these methods is that they promote early germination leading to improved germination index, seedling vigor, increased shoot and radicle length and a decrease in detrimental seed microflora^8^.

Among industrially important microalgae species, *Chlorella* spp., *Nannochlorpsis* spp., and *Arthrospira platensis* (*Spirulina platensis*.) have been used as a renewable source of food, nutraceuticals, animal feed, and agrochemicals^9,10^. *Chlorella* spp. (Chlorophyta) is a green unicellular microalga with biological and pharmacological properties. *Chlorella vulgaris* is a rich source of macro- and micronutrients, proteins, lipids, carbohydrates, pigments, and antioxidant metabolites^11^, and contains significant levels of growth-promoting factors, including cytokinins such as iso-pentenyladenine, zeatin, and their conjugated ribosides^12^. *Nannochloropsis* spp. (Eustigmataceae) is a genus of a unicellular, marine microalga, characterized by high lipid productivity, especially triacylglycerol (TAG) and omega-3 long-chain polyunsaturated fatty acid such as eicosapentaenoic acid^13^. Microalgae are considered as valuable sources of essential vitamins (including vitamins A, E, C, B_1_, and B_12_), carbohydrates, bio-active acids (e.g., folic, pantothenic, and nicotinic), and microelements^14^. *Arthrospira platensis* (commonly known as *Spirulina*) (Cyanophyta) is filamentous blue - green cyanobacteria widely utilized for their protein content, carbohydrates, essential minerals including copper, iron, and magnesium. *Spirulina* also contains B-complex vitamins, antioxidants and compounds with antibacterial and antifungal properties, further supporting its role as an effective bio stimulant in agricultural systems^15^.

In a recent study, microalgae cell suspension and extracts were successfully applied as bio stimulants in seed treatments of several crops, such as priming of seeds of tomato, and cucumber seeds with *C. vulgaris*, and the consortium consisting of Chlorella spp., Spirulina spp., Scenedesmus spp., and Synechocystis spp., resulted in a higher germination rate compared to untreated seeds^9,16^. Extracts of *C. vulgaris*, and *Scenedesmus quadricauda* act as bio stimulants in the early stages of sugar beet cultivation, improving growth and modulating gene expression related to nutrient acquisition^17^, improve seed germination, faster cotyledon emergence, and seedling weight in spinach^18^. Pre-soaking sugar beet seeds in filtrates of *Arthrospira platensis*, *Anabaena oryzae*, *Oscillatoria* spp., and *Nostoc muscorum* for 24 h led to significantly higher germination percentages compared to water-soaked controls^19^. Priming milkweed (*Calotropis procera* Ait.) seeds with 5 mL L^− 1^ extracts of *Arthrospira platensis* and *Sargassum angustifolium* significantly improved germination percentage, vigor index, seedling length, and biomass under both normal and saline conditions. Conversely, higher extract concentrations (> 10 mL L^− 1^) exerted inhibitory effects on these parameters^20^. Similarly, priming dwarf pea seeds with the microalgae *Desmodesmus* sp. MAS1 and *Heterochlorella* sp. MAS3 improved rhizosphere health, resulting in increased plant growth and productivity^21^.

Thus, algal extracts may exert a biphasic effect, emphasizing the need for dosage optimization. However, comparative, dose-dependent evaluations of *C. vulgaris*,* N. salina*, and *A. platensis* on common bean germination, integrating growth and biochemical responses, remain limited. The present study addresses this gap by systematically assessing the effects of the three microalgae species on seed germination, seedling vigor, and key biochemical indicators.

## Materials and methods

### Materials

Bronco variety of common bean seeds (*Phaseolus vulgaris* L.). were provided by Horticulture Research Institute, Agriculture Research Center, Ministry of Agriculture, Giza, Egypt. Dry, pure algal samples of C. vulgaris, N. salina and *A. platensis* were provided by the Algal Biotechnology Unit, National Research Centre, Dokki, Egypt.

### Preparation of algal extracts

The method described by Pant et al.,^22^ was employed to extract 5 g air-dried powder from Chlorella vulgaris, Nannochloropsis salina and *Arthrospira platensis* algae using 100mL 80% methanol (PIOCHEM) at 60 °C for 24 h under continuous stirring, followed by filtration of the extract. To ensure biological compatibility and avoid phytotoxic effects, methanol was completely removed via rotary evaporation at 60 °C under reduced pressure. The obtained dry residue was then re-dissolved in distilled water. Because of their adaptability and efficiency in extracting a wide range of natural compounds, water-methanol solvent systems are widely used. Polar organic solvents like methanol are very good in dissolving polar and semi-polar substances like terpenoids, alkaloids, flavonoids, and phenolic acids^23^.

To allow for a meaningful comparison among the three algal species, the extracts were standardized using a defined weight-to-volume (w/v) ratio to achieve the target concentration, ensuring that the bioactivity observed was attributable to the concentration of the extracted compounds rather than to solvent effects or differences in extraction efficiency.

### Analyses of algae

Total soluble sugar (TSS) was estimated as described by Umbreit et al.,^24^, and the procedure of Lowry et al.,^25^ was followed for total soluble protein contents (TSP) in air dry samples of C. vulgaris, N. salina, and A. platensis microalgal.

#### Plant materials and treatments

Common bean seeds were surface sterilized using a 2.5% sodium hypochlorite solution for 3 min and then rinsed with distilled water. The seeds were divided into three batches, and each batch was further divided into 7 or 6 groups of 100 seeds. These groups (19) were soaked in different solutions. The soaking process involved placing fixed number of seeds from each batch into glass containers containing 100 mL treatment solutions (*C. vulgaris*, *N. salina*, and *A. platensis*) extracts at concentrations of 0.13, 0.25, 0.5, 1.0, 2.0 and 5.0%, distilled water was used for the control treatment, for 4 h at 25 °C.

Following this, the seeds of the control and each treatment were washed thoroughly with distilled water, then transferred to small pots (20 cm long, 15 cm wide, and 10 cm depth), containing sterilized sandy soil for 6 days. According to ISTA rules, seedling measurements were taken after 6 days at optimal temperature. The experiment involved the replication of each treatment (19) six times. A total of 114 pots (19*6), each consisting of 20 seeds, were utilized. Furthermore, all pots were carefully positioned within a growth chamber at 56% relative humidity, and a temperature range of 25–30 °C, with alternating periods of darkness and light (14/10 h), light intensity (25 µmol m^− 2^ s^− 1^). These conditions were maintained consistently to enable controlled experimentation.

### Growth measurement

#### Germination indices

The germination percentage (GP) was calculated as a percentage of total germinated seeds on 6 days after soaking (DAS), according to the following:

$$\:\mathrm{G}\mathrm{e}\mathrm{r}\mathrm{m}\mathrm{i}\mathrm{n}\mathrm{a}\mathrm{t}\mathrm{i}\mathrm{o}\mathrm{n}\:\mathrm{p}\mathrm{e}\mathrm{r}\mathrm{c}\mathrm{e}\mathrm{n}\mathrm{t}\mathrm{a}\mathrm{g}\mathrm{e}\:\left(\mathrm{G}\mathrm{P}\right)\:=\:\frac{n}{N}\times\:100$$

where n is number of germinated seeds, and N is total number of seeds for bioassay.

The seedling vigor index (SVI) was measured at 6 days old seedling, according to Noorhossein et al.^26^ using the following formula:

$$\:\mathrm{S}\mathrm{V}\mathrm{I} =\mathrm{l}\mathrm{e}\mathrm{n}\mathrm{g}\mathrm{t}\mathrm{h}\:\mathrm{o}\mathrm{f}\:\mathrm{s}\mathrm{e}\mathrm{e}\mathrm{d}\mathrm{l}\mathrm{i}\mathrm{n}\mathrm{g}\:\times\:\mathrm{G}\mathrm{P}$$

A minimum of 12 randomly selected 6-day-old seedlings from both control and treatment groups were used to evaluate growth parameters, including plumule and radicle length (cm), as well as fresh and dry weights (g) per seedling. The samples were oven-dried at 75 °C until a constant weight was reached, after which they were analyzed for total soluble sugars and total soluble proteins. Fresh samples were also used to determine fresh weight and to assess enzymatic activities.

#### Sample extraction for hydrolytic enzymes

Two grams of fresh common bean seedlings were homogenized in a pre-chilled mortar and pestle with 10 mL of double-distilled water until a uniform paste was obtained. The homogenate was then centrifuged at 1252 × g at 4 °C for 20 min. The resulting clear supernatant was collected and used for subsequent enzymatic assays.

#### α-amylase enzyme (EC 3. 2.11)

α-Amylase activity in common bean seedlings was determined according to Bergmeyer^27^. The reaction mixture consisted of 0.5 mL starch prepared in phosphate buffer (pH 7.0), 0.5 mL double-distilled water, and 0.5 mL enzyme extract. After incubation at 25 °C for 10 min, 1 mL of color reagent (1% dinitrosalicylic acid) was added, and the mixture was boiled in a water bath for 10 min. It was then cooled in an ice bath, and the final volume was adjusted to 10 mL with distilled water. The absorbance was measured at 546 nm using a Cecil CE 1010 spectrophotometer.

#### Protease enzyme (EC 3.4.21.40)

Protease activity was determined according to the method of Bergmeyer^27^. The reaction mixture consisted of 1 mL of 1% casein prepared in phosphate buffer (pH 7.5) and 1 mL of enzyme extract. After incubation at 37 °C for 1 h, the reaction was terminated by adding 2 mL of 10% trichloroacetic acid. The mixture was then centrifuged at 1252 × g and 4 °C for 20 min. The amino acid content in the supernatant was estimated following the method of Lowry et al.,^25^ to evaluate protease activity based on casein hydrolysis.

#### Total soluble sugars and total soluble proteins extraction

A 0.1 g sample of common bean seedlings was homogenized in 5 mL of 70% ethanol. The homogenate was then centrifuged at 2817 × g for 15 min, and the resulting supernatant was diluted to a final volume of 15 mL with distilled water.

#### Total soluble sugars

Total soluble sugars (TSS) were determined using the anthrone method as described by Umbreit et al.,^24^. Six milliliters of anthrone reagent (2 g L⁻¹ in 95% H₂SO₄) were added to 3 mL of the sample, and the mixture was heated in a boiling water bath for 3 min. After cooling, the developed color was measured spectrophotometrically at 620 nm. The results were expressed as milligram equivalents of glucose per milligram of dry weight. A calibration curve was constructed using glucose standards at concentrations of 10, 20, 30, 40, and 50 ppm.

#### Total soluble proteins

The procedure described by Lowry et al.,^25^ was followed to determine total soluble proteins. Briefly, 1 mL of common bean extract was mixed with 5 mL of a freshly prepared solution (50:1, v/v) consisting of 2% sodium carbonate in 0.4% sodium hydroxide and 0.5% copper sulfate in 1% (w/v) sodium tartrate. The mixture was allowed to stand for 10 min, after which 0.5 mL of Folin reagent was added. The optical density was measured spectrophotometrically at 750 nm after 30 min. The results were expressed as milligram equivalents of bovine serum albumin per milligram of dry weight. A calibration curve was prepared using standard concentrations of 100, 200, 300, 400, and 500 ppm.

### Statistical analysis

The data were expressed as the mean of six replicates, with each replicate consisting of two seedlings used to evaluate growth parameters, including germination indices and seedling development. Enzyme activity, total soluble sugars, and total soluble proteins were calculated as the mean of three replicates. Statistical analysis was performed using one-way analysis of variance (ANOVA). Differences among means were assessed using Duncan’s Multiple Range Test. All analyses were conducted using IBM SPSS Statistics for Windows (Version 21), and differences were considered statistically significant at *P* ≤ 0.05.

## Results

### Germination percentage

**Fig. 1:**
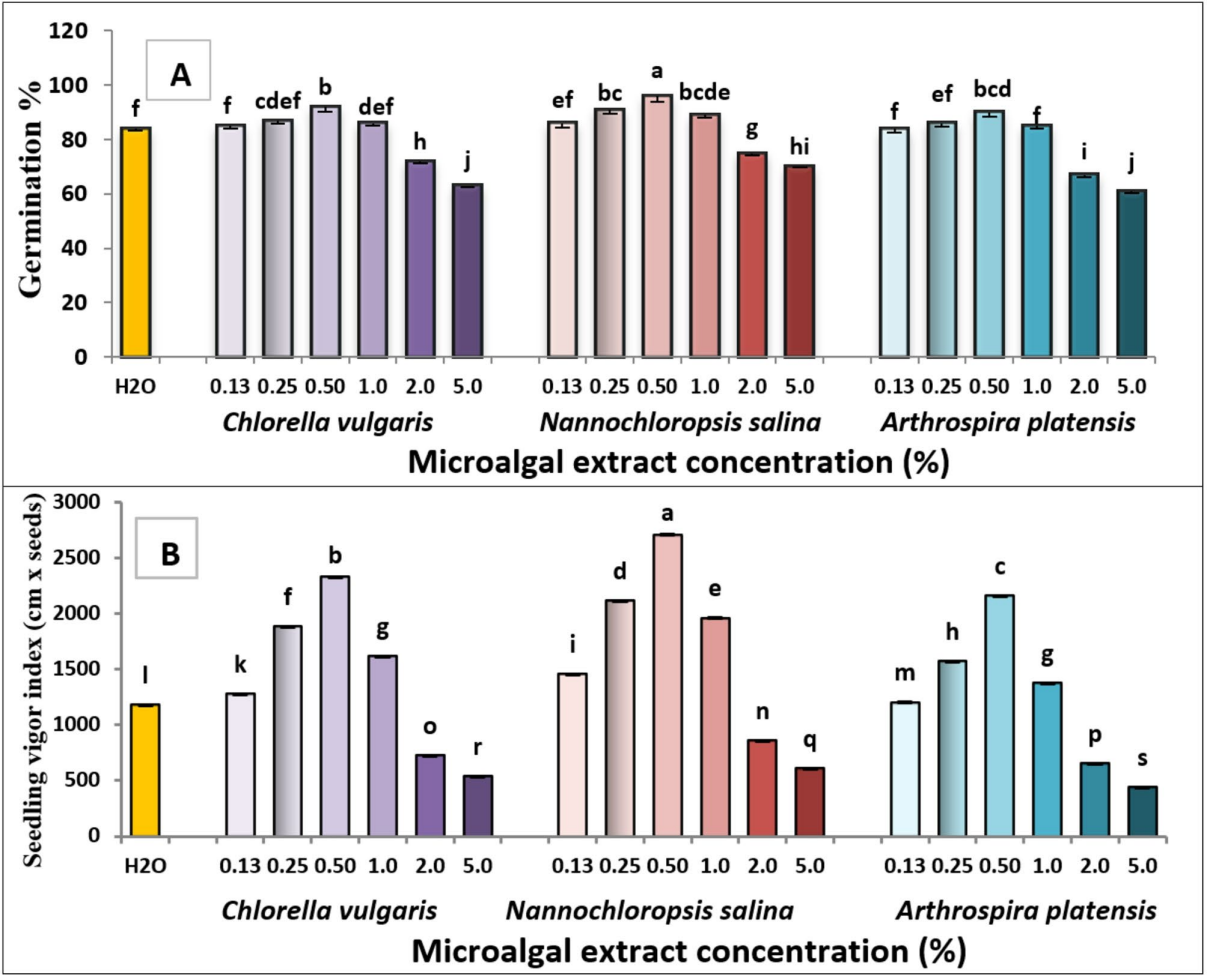
Changes in (**A**) germination percentage and (**B**) seedling vigor index (SVI) of 6 day- old seedlings of Bronco variety as affected by seed presoaking for 4 h in different concentrations of *Chlorella vulgaris*, *Nannochloropsis salina*, and *Arthrospira platensis* extracts at 0.0, 0.13, 0.25, 0.5, 1.0, 2.0%, and 5.0% and grown at 25–30 °C. The data are presented as mean of 6 replicates (each of 20 seeds) ± SE. Different letters indicate significant differences between treatments (Duncan test, *P* ≤ 0.05). Means, in each column, followed by similar letters are not significantly different.

Soaking common beans seeds for 4 h in various concentrations (0.13, 0.25, 0.5, and 1.0%) of *C. vulgaris*, *N. salina*, and *A. platensis* extracts increased germination percentage compared to the control. Germination percentage gradually declined at higher concentrations (Fig. 1A). The highest germination percentage (96, 92, and 90%) occurred when seeds were soaked in solutions containing 0.5% of *Nannochloropsis*, *Chlorella*, and *Arthrospira*, respectively in comparison with 84% for the control. Conversely, germination percentage was negatively impacted at higher concentrations (2.0, and 5.0%), recording − 27.38, − 25.00, and − 16.67% decline at 5.0% of *Arthrospira*, *Chlorella*, and *Nannochloropsis*, respectively in comparison to the control treatment (Fig. 1A).

### Seedling vigor index

All algal extract treatments up to 1.0% had a positive impact on SVI values compared to control, consistent with germination percentages with the optimum at 0.5%. Among these treatments, 0.5% concentration of *N. salina* followed by *C. vulgaris*, and then *A. platensis* extracts, resulted in the most significant increase in SVI, with values approximately 130.20%, 97.93%, and 83.67% higher than control, respectively. However, SVI gradually declined at higher concentrations of algal extracts (2.0% and 5.0%). *A. platensis* at 5.0% induced the largest decrease in SVI (-62.65% lower than control), followed by *C. vulgaris*, and *N. salina* (-54.46% & -48.21% respectively) (Fig. 1B).

### Seedling growth

**Fig. 2:**
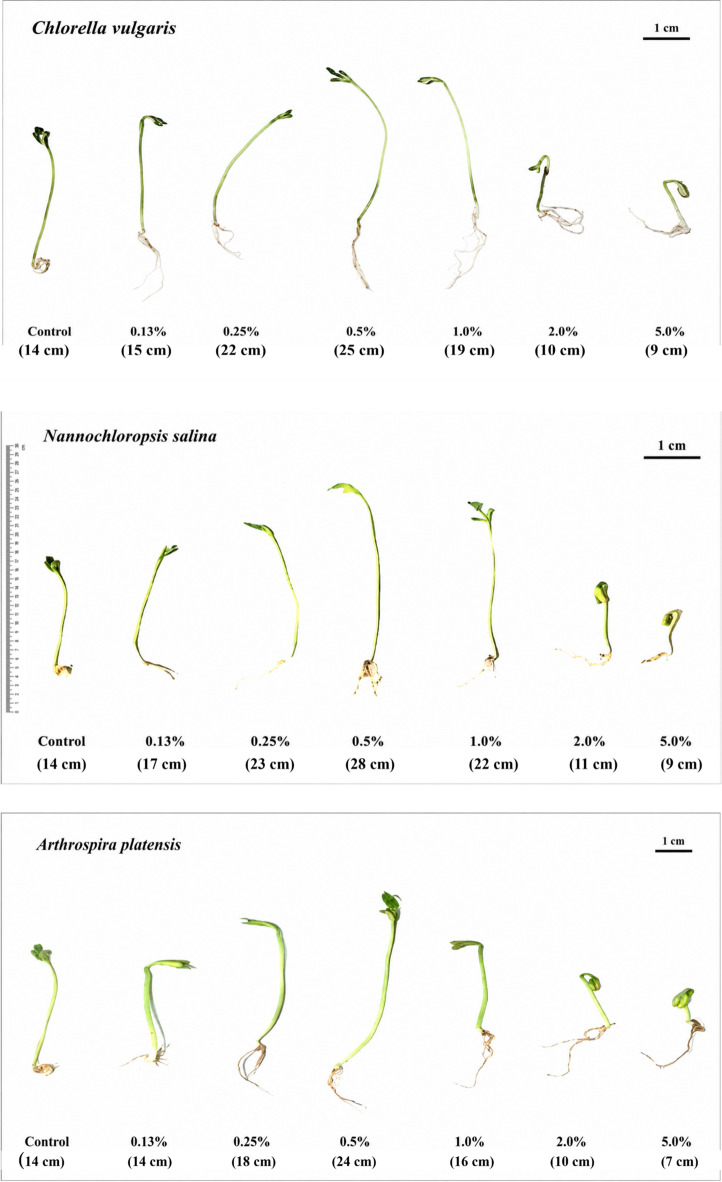
Seedlings growth of Bronco variety as affected by seed presowing for 4 h in *Chlorella vulgaris*, *Nannochloropsis salina*, and *Arthrospira platensis* extracts at 0.0, 0.13, 0.25, 0.5, 1.0, 2.0, and 5.0% followed by 6 days germinated at 25–30 °C.

**Fig. 3:**
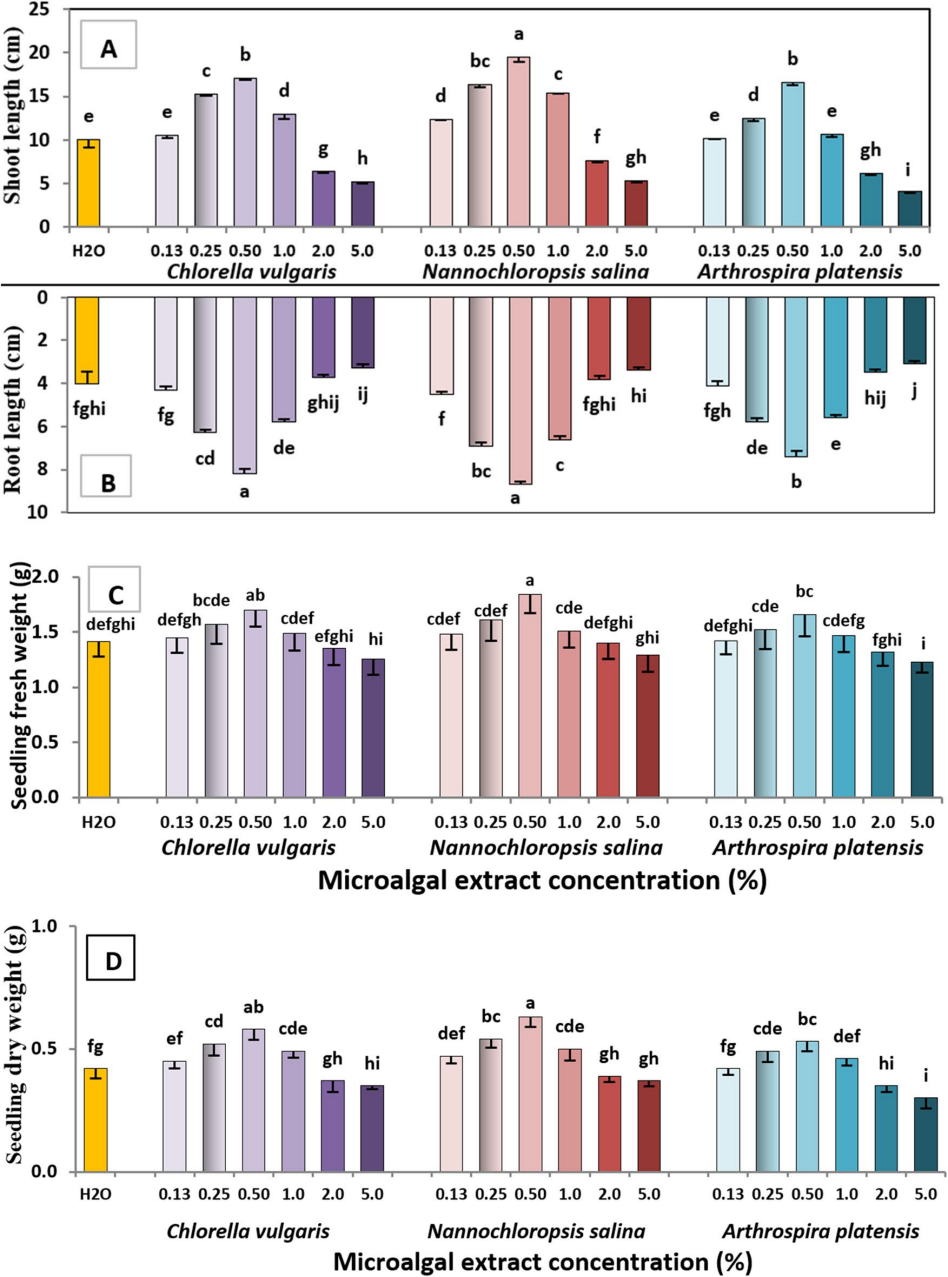
(**A**–**D**) Growth criteria of seedlings of Bronco variety as affected by presoaking for 4 h in different concentrations of *C. vulgaris*, *N. salina*, and *A. platensis* extracts at 0.0, 0.13, 0.25, 0.5, 1.0, 2.0%, and 5.0% and grown at 25–30 °C. (**A**) Shoot length, (**B**) Root length, (**C**) seedling fresh weight and (**D**) Seedling dry weight. The data are presented as mean of 6 replicates (each of 2 seedlings). Vertical bars represent ± SE. Different letters indicate significant differences between treatments (Duncan test, *P* ≤ 0.05). Means, in each column, followed by similar letters are not significantly different.

Shoot and root length in common bean increased when seeds were pretreated with extracts of *Nannochloropsis*, *Chlorella*, and *Arthrospira* at concentrations ranging from 0.13 to 1%, compared to control seedlings (Figs. 2 and 3A,B). The most significant results occurred when seeds were soaked in a solution containing 0.5% *Nannochloropsis*, followed by *Chlorella*, and then *Arthrospira* at the same concentration. *Nannochloropsis* extract was more effective in promoting the shoot and root lengths in common bean seedlings, than *Chlorella* and *Arthrospira* at comparable concentrations (Fig. 3A,B). The three extracts at a concentration of 5.0% significantly reduced shoot length by − 59.00%, − 48.00%, and − 47.00%, respectively in comparison with their respective controls. These extracts also decreased root length by − 22.50%, − 17.50%, and − 15.00%, and had a negative impact on seedling fresh weight, with reductions of − 12.77%, − 10.64%, and − 8.51%. Similarly, seedling dry weights were negatively affected by -28.57%, -16.67%, and − 11.90% at 5.0% level (Fig. 3C,D).

### Activities of α-amylase and protease enzymes

**Fig. 4:**
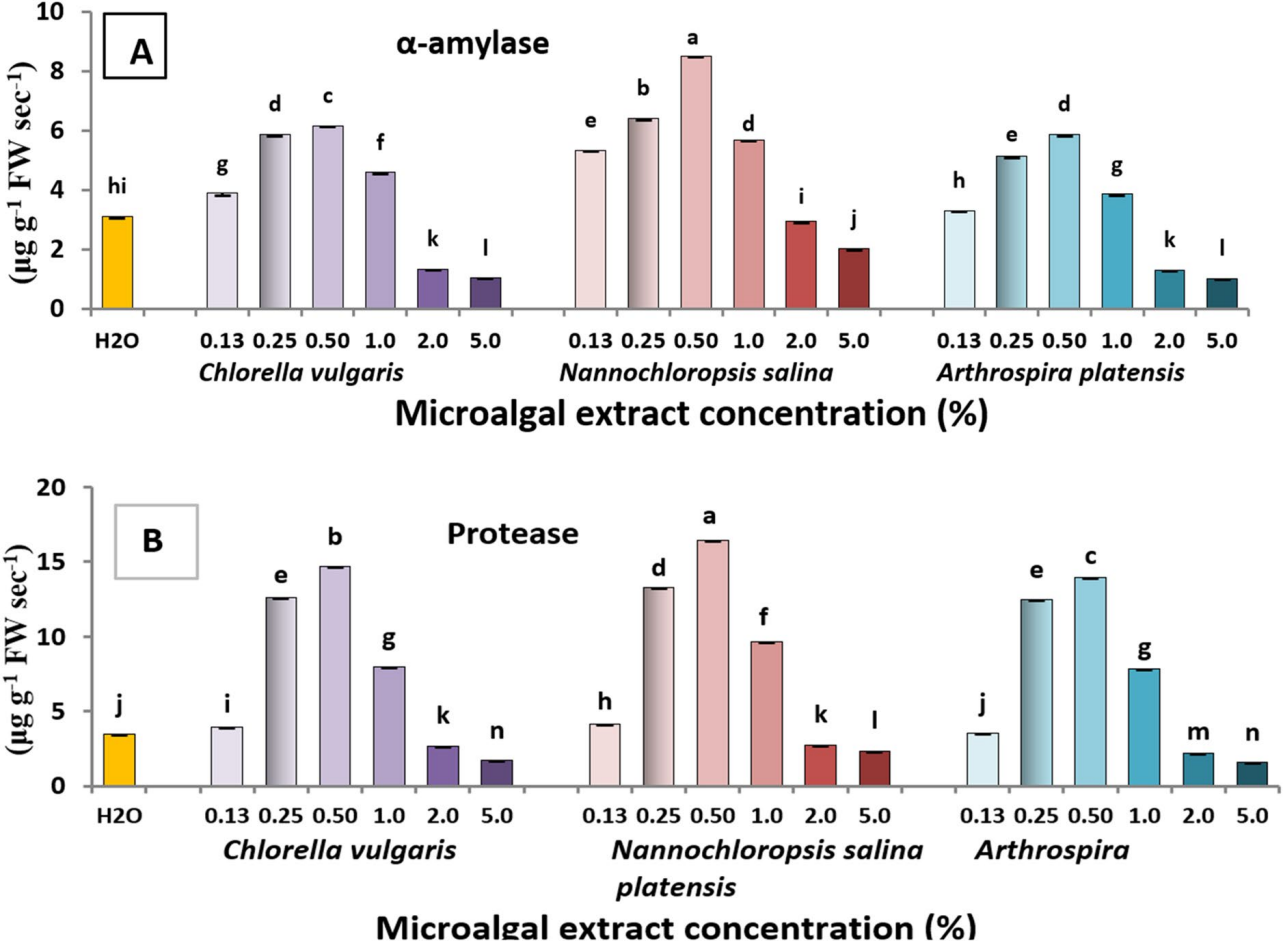
Changes in enzymatic activities of (**A**) α-amylase and (**B**) protease (µg g^− 1^ FW sec^− 1^) of 6 day-old seedlings of Bronco variety as affected by seed presoaking for 4 h in different concentrations of *C. vulgaris*, *N. salina*, and *A. platensis* extracts at 0.0, 0.13, 0.25, 0.5, 1.0, 2.0%, and 5.0% and grown at 25–30 °C. The data are presented as mean of 3 replicates. Vertical bars represent ± SE. Different letters indicate significant differences between treatments (Duncan test, *P* ≤ 0.05). Means, in each column, followed by similar letters are not significantly different.

Soaking common bean seeds in *C. vulgaris*, *N. salina* and *A. platensis* extracts at concentrations of 0.13% to 1.0% for duration of 4 h resulted in increased activity of α-amylase and protease enzymes in 6-day-old common bean seedlings, compared to the control group. These enzyme activities showed a progressive decrease at higher concentrations (Fig. 4). Furthermore, the use of *N. salina* at comparable concentrations was more effective than *C. vulgaris* and *A. platensis* in enhancing the activities of α-amylase and protease enzymes in common bean seedlings. The highest activities of α-amylase and protease enzymes were obtained when the seeds were soaked in 0.5% solutions of *Nannochloropsis* being (8.50 and 16.43 µg g^− 1^ F w sec^− 1^), followed by *Chlorella* 6.16, and 14.69 µg g^− 1^ F. w sec^− 1^), then *Arthrospira* (5.86, and 13.93 µg g^− 1^ F. w sec^− 1^), compared with (3.11, and 3.46 µg g^− 1^ F. w sec^− 1^) in control common bean seedling, respectively.

The lowest activities of α-amylase and protease enzymes were achieved when seeds were soaked in solutions with a concentration of 5.0% of *Arthrospira*, *Chlorella*, and *Nannochloropsis* (Fig. 4).

### Total soluble sugars and total soluble proteins

Soaking common bean seeds in various algal (*Nannochloropsis*, *Chlorella*, *Arthrospira*) extracts at 0.13, 0.25, 0.5, and 1.0% concentrations led to an increase in the levels of total soluble sugars (TSS) and total soluble proteins (TSP) in the common bean seedlings. The highest significant TSS (40.73, 36.96, and 34.63 mg g^− 1^ FW) and TSP (42.43, 41.53, and 40.19 mg g^− 1^ FW) levels occurred when the seeds were soaked in solutions containing *Nannochloropsis*, *Chlorella*, or *Arthrospira* at a concentration of 0.5%, compared with (30.46, and 36.05 mg g^− 1^ FW) for their respective controls (Table 1). As the concentrations surpassed 1.0%, there was a gradual decrease in TSS and TSP values. The lowest TSS (13.36, 14.47, and 16.92 mg g^− 1^ FW) and TSP (18.77, 19.32, 20.39 mg g^− 1^ FW) values occurred when seeds were soaked in solutions with a concentration of 5.0% of *Arthrospira*, *Chlorella*, and *Nannochloropsis*, respectively (Table 1).

**Table 1:** Soluble sugars and soluble proteins (mg g^− 1^ DW) in extracts of *Chlorella vulgaris*,* Nanochloropsis salina*, and *Arthrospira platensis*, as well as in 6 day-old seedlings of Bronco variety as affected by seed presoaking for 4 h in either *C. vulgaris*, *N. salina*, or *A. platensis* extracts at 0.0, 0.13, 0.25, 0.5, 1.0, 2.0, and 5.0% and grown at 25–30 °C.

Treatments	Soluble sugar	Soluble protein
	mg g^− 1^ DW	mg g^− 1^ DW
*Chlorella vulgaris*	1.23 ± 0.02	11.20 ± 0.12
*Nanochloropsis salina*	1.39 ± 0.04	17.00 ± 0.17
*Arthrospira platensis*	1.05 ± 0.03	8.59 ± 0.05
Control (H_2_O)	30.46^g^ ±0.42	36.05^f^ ±0.15
*C. vulgaris* 0.13%	33.68^efg^ ±0.38	37.08^e^ ±0.18
*C. vulgaris* 0.25%	33.79^b^ ±0.57	41.02^e^ ±0.16
*C. vulgaris* 0.5%	36.96^b^ ±0.27	41.53^b^ ±0.28
*C. vulgaris* 1.0%	29.58^d^ ±0.26	38.89^g^ ±0.33
*C. vulgaris* 2.0%	17.80^i^ ±0.13	23.22^i^ ±0.10
*C. vulgaris* 5.0%	14.47^l^ ±0.13	19.32^k^ ±0.17
*N. salina* 0.13%	34.74^e^ ±0.13	37.70^d^ ±0.27
*N. salina* 0.25%	36.79^b^ ±0.14	41.19^b^ ±0.28
*N. salina* 0.5%	40.73^a^ ±0.36	42.43^a^ ±0.39
*N. salina* 1.0%	35.85^c^ ±0.14	39.95^c^ ±0.30
*N. salina* 2.0%	19.47^h^ ±0.12	25.46^h^ ±0.29
*N. salina* 5.0%	16.92^k^ ±0.11	20.39^j^ ±0.19
*A. platensis* 0.13%	30.13^fg^ ±0.27	36.67^fg^ ±0.25
*A. platensis* 0.25%	33.24^d^ ±0.14	38.56e ± 0.24
*A. platensis* 0.5%	34.63^c^ ±0.47	40.19^d^ ±0.36
*A. platensis* 1.0%	29.41^ef^ ±0.30	37.29^g^ ±0.32
*A. platensis* 2.0%	16.92^j^ ±0.18	21.90^j^ ±0.22
*A. platensis* 5.0%	13.36^l^ ±0.17	18.77^l^ ±0.19
L.S.D at 5%	0.83	0.81

Each result is a mean of 3 replicates ± SE. Different letters indicate significant differences between treatments (Duncan test, *P* ≤ 0.05). Means, in each column, followed by similar letters are not significantly different.

## Discussion

Treatments of *Arthrospira*, *Chlorella*, and *Nannochloropsis* at low concentrations enhanced germination percentage, seedling vigor index, and the main growth criteria of common beans seedlings. This enhancement is linked to the seed pretreatment process, which regulates moisture uptake, facilitating the hydration of the seed coat. This hydration process softens the seed coat, allowing for increased water absorption rates, thereby easing the radicle’s breakthrough and initiating growth. Seed priming agents, including bio stimulant compounds, enhance physiological processes within seed embryos prior to germination^28^. Pretreatment method also aids in the initial growth and emergence of the radicle, accompanied by associated biochemical activities^17^. Acceleration of germination time, growth and development has been largely attributed to the presence of natural growth regulators, such as kinetin, ethylene, and gibberellic acid in algal extracts^29^. Low concentration of *N. salina* especially at 0.5% significantly enhanced shoot and root elongation and biomass accumulation,, likely due to its higher levels of gibberellins and indole-3-acetic acid in algal extract^10^.

High concentrations of algal extracts inhibited seed germination and seedling growth this may be due to oxidative stress and hormonal imbalance. Comparable results have been reported previously by Godlewska et al.,^30^, who reported the negative impact of applying *S. platensis* extract on radish fresh weight at concentrations exceeding 15%. This inhibition may be attributed to supra-optimal phytohormone levels and increased osmotic pressure, which switches the plant’s response from growth promotion to stress mitigation. Similar observations were reported by Barone et al.,^17^, highlighting variability in the protein, lipid, carbohydrate, pigment, and elemental composition of algal extracts. Elevated levels of growth hormones can inhibit root elongation and overall plant development as reported by Gharib et al.,^10^ for gibberellic acid and auxin content in *Chlorella vulgaris*, *Nanochloropsis salina*, and *Arthrospira platensis*.

The activities of α-amylase and protease enzymes in common bean seedlings are significantly elevated with increasing concentrations of three microalgal extracts, up to 1.0%. Enhanced α-amylase activity promoted efficient starch hydrolysis into soluble sugars, while elevated protease activity facilitated the mobilization of storage proteins, collectively supporting higher respiration rates, increased ATP production, and accelerated seedling growth. Seed priming leads to increased synthesis of germination-promoting substances, membrane reorganization, and enhanced hydrolytic enzyme activity^31^. Gibberellic acid (GA) growth promotor plays a central role in regulating α-amylase synthesis in the aleurone layer^32,33^. In contrast, the activity of α-amylase and protease enzymes was significantly diminished when the seeds were soaked in solutions containing 5.0% concentration of A. platensis, C. vulgaris and N. salina extracts. This inhibition may be due to an increased osmotic potential that restricts water imbibition and delays metabolic activation, as reported by Bewley et al.^34^. Elevated levels of microalgal metabolites may suppress GA biosynthesis or promote abscisic acid (ABA) accumulation, thereby inhibiting gene expression of the enzyme.

Previous studies have documented variations in the protein, lipid, carbohydrate, pigment, and elemental contents of algal extracts^11,15^. During the germination process, enzymes like amylases, proteases, and lipases break down stored substances, producing compounds essential for seedling development^35,36^. Seeds treated with algal cellular extracts exhibit superior germination rates, plant growth, and concentrations of soluble carbohydrates, proteins, and free amino acids compared to untreated seeds^37,38^. The application of *C. vulgaris* suspension on foliage and soil enriched with N-urea resulted in the most substantial increases in leaf and seed total protein and carbohydrate contents in *P. vulgaris*, relative to the control group^39^. A comparable rise in protein content was observed in lettuce seedlings’ shoots and roots treated with *Tetradesmus quadricauda* extract^40^. At high concentrations, the osmotic potential of the solution may generate a strong osmotic gradient that restricts water uptake by the seeds. Insufficient imbibition limits the activation of metabolic processes required for starch hydrolysis into soluble sugars and for protein biosynthesis, effectively inducing a condition analogous to chemical drought. Consistent with these findings, treating *Lupinus luteus* plants with a 1.0% *S. platensis* extract adversely impacted both insoluble and total carbohydrate content^41^.

## Conclusion

This study demonstrates that microalgal extracts can serve as promising natural alternative to synthetic agrochemicals. Seed priming of common bean with extracts from *C. vulgaris*,* N. salina*, and *A. platensis*, particularly at a concentration of 0.5% markedly enhanced seed germination, seedling growth, hydrolytic enzyme activities, and the accumulation of total soluble sugars and proteins compared with untreated controls. Among the tested species, *Nannochloropsis salina* exhibited the most pronounced bio stimulatory effect. In contrast, higher extract concentrations exerted inhibitory effects, highlighting the importance of dose optimization.

The findings highlight the potential application of microalgal extracts as eco-friendly seed-priming agents that could complement or partially replace conventional chemical treatments in sustainable agricultural systems. Nevertheless, validation under field conditions remains essential. Future research should focus on large-scale field trials, comprehensive metabolomic profiling of microalgal extracts to identify the key bioactive compounds, and the evaluation of their effectiveness across different crop species and environmental conditions.

## Data Availability

All data generated or analyzed during this study are included in this article.
